# Axitinib in Combination With Toripalimab, a Humanized Immunoglobulin G_4_ Monoclonal Antibody Against Programmed Cell Death-1, in Patients With Metastatic Mucosal Melanoma: An Open-Label Phase IB Trial

**DOI:** 10.1200/JCO.19.00210

**Published:** 2019-08-12

**Authors:** Xinan Sheng, Xieqiao Yan, Zhihong Chi, Lu Si, Chuanliang Cui, Bixia Tang, Siming Li, Lili Mao, Bin Lian, Xuan Wang, Xue Bai, Li Zhou, Yan Kong, Jie Dai, Kai Wang, Xiongwen Tang, Huaning Zhou, Hai Wu, Hui Feng, Sheng Yao, Keith T. Flaherty, Jun Guo

**Affiliations:** ^1^ Peking University Cancer Hospital and Institute, Beijing, People’s Republic of China; ^2^ OrigiMed, Shanghai, People’s Republic of China; ^3^ Shanghai Junshi Biosciences, Shanghai, People’s Republic of China; ^4^ Cancer Center, Massachusetts General Hospital, Boston, MA

## Abstract

**PURPOSE:**

Metastatic mucosal melanoma responds poorly to anti–programmed cell death-1 (PD-1) monotherapy. Vascular endothelial growth factor (VEGF) has been shown to play an important immunosuppressive role in the tumor microenvironment. The combination of VEGF inhibition and PD-1 blockade provides therapeutic opportunities for patients refractory to either therapy alone.

**PATIENTS AND METHODS:**

We conducted a single-center, phase IB trial evaluating the safety and preliminary efficacy of toripalimab, a humanized immunoglobulin G_4_ monoclonal antibody against PD-1 in combination with the VEGF receptor inhibitor axitinib in patients with advanced melanoma, including patients with chemotherapy-naïve mucosal melanomas (88%). Patients received toripalimab at 1 or 3 mg/kg via intravenous infusion every 2 weeks, in combination with axitinib 5 mg orally twice a day, in a dose-escalation and cohort-expansion study until confirmed disease progression, unacceptable toxicity, or voluntary withdrawal. The primary objective was safety. Secondary objectives included efficacy, pharmacokinetics, pharmacodynamics, immunogenicity, and tumor tissue biomarkers.

**RESULTS:**

Thirty-three patients were enrolled. No dose-limiting toxicities were observed. Ninety-seven percent of patients experienced treatment-related adverse events (TRAEs). The most common TRAEs were mild (grade 1 or 2) and included diarrhea, proteinuria, hand and foot syndrome, fatigue, AST or ALT elevation, hypertension, hypo- or hyperthyroidism, and rash. Grade 3 or greater TRAEs occurred in 39.4% of patients. By the cutoff date, among 29 patients with chemotherapy-naïve mucosal melanoma, 14 patients (48.3%; 95% CI, 29.4% to 67.5%) achieved objective response, and the median progression-free survival time was 7.5 months (95% CI, 3.7 months to not reached) per Response Evaluation Criteria in Solid Tumors (RECIST) version 1.1.

**CONCLUSION:**

The combination of toripalimab plus axitinib was tolerable and showed promising antitumor activity in patients with treatment-naïve metastatic mucosal melanoma. Patients enrolled in this study were all Asian, and this combination therapy must be validated in a randomized phase III trial that includes a non-Asian population before it can become a standard of care.

## INTRODUCTION

Mucosal melanoma is a rare melanoma subtype, composing approximately 1.3% of all melanomas in white populations.^1^ In contrast, it is the second most common subtype in Asian populations, constituting 22% to 25% of all melanomas in Asian patients.^2,3^

Compared with chronic ultraviolet exposure–associated cutaneous melanoma, mucosal melanoma is a more aggressive malignancy with lower tumor mutational burden (TMB)^4^ and poorer responses to therapies.^5-7^ A genome-wide mutational landscape study has shown that, in contrast to heavily mutated ultraviolet-induced cutaneous melanoma, mucosal melanomas harbor unique mutations with unknown etiology,^4^ which provides a molecular basis for the discordant clinical treatment results of melanoma in Asian versus white populations. Curtin et al^8,9^ reported infrequent *BRAF* mutations in mucosal melanomas (11%) but frequent *BRAF* mutations in cutaneous melanomas unrelated to chronic sun-induced damage (non-CSD; 59%), whereas *KIT* amplifications or activating mutations were more common in mucosal melanomas (39%) than non-CSD melanomas (0%).^9^ However, two large-scale studies on *BRAF* and *KIT* mutations in Chinese patients found a similar frequency of *BRAF* mutations (12.5%) but a lower frequency of *KIT* aberrations (20.1%) in patients with mucosal melanoma compared with white patients.^10,11^ A retrospective study involving 12 patients with mucosal melanoma harboring *BRAF* mutations demonstrated a median progression-free survival (PFS) time of 4.4 months and median overall survival (OS) time of 8.2 months, with an overall response rate (ORR) of 20.0%, after treatment with BRAF inhibitors.^12^ Several phase II trials included patients with mucosal melanoma to evaluate the efficacy of a KIT inhibitor in patients with *KIT* aberrations. The results were unsatisfactory regardless of race, with an ORR of 16.0% to 23.3% and a median PFS of only 2.8 to 3.7 months.^13-15^ In addition, in a large cohort study (N = 522), the median OS of patients with mucosal melanoma was significantly shorter than that of patients with nonmucosal melanoma (3.58 *v* 4.67 years, respectively), indicating an unmet need for effective systemic treatments for the mucosal subtype.^3^

Immune checkpoint inhibitors have improved the outcomes of advanced melanoma, but the benefits are mainly manifested in patients with the cutaneous subtype rather than mucosal subtype. The combination of ipilimumab and anti–programmed cell death-1 (PD-1) inhibitors seems to improve outcomes compared with monotherapy in mucosal melanoma. However, the data regarding immunotherapy among Chinese patients are limited. The KEYNOTE-151 study (ClinicalTrials.gov identifier: NCT02821000) showed a 13.3% ORR with pembrolizumab in Chinese patients with mucosal melanoma refractory to chemotherapy.^16^ However, a phase II trial of toripalimab, also known as JS001 or TAB001, a humanized immunoglobulin G_4_ monoclonal antibody against PD-1,^17^ in 128 pretreated Chinese patients with advanced melanoma showed a higher ORR for patients with CSD (35.3%) and non-CSD (33.3%) subtypes than for patients with the mucosal subtype (0%).^18^

A previous clinical study demonstrated that vascular endothelial growth factor (VEGF) expression level was associated with poor outcomes in patients with mucosal melanoma.^19^ However, antiangiogenic therapy alone has not shown significant improvement compared with chemotherapy in melanoma.^20^ In addition to its role in vascular growth, VEGF has also emerged as an important immunosuppressive agent in the tumor microenvironment.^21,22^ In vivo studies have shown that angiogenesis inhibition,^23^ specifically simultaneous inhibition of the VEGF receptor (VEGFR) and PD-1 pathways in a mouse model, increased T-cell infiltration and suppressed tumor growth synergistically.^24^

Toripalimab has shown preliminary clinical activity in both phase I and phase II trials in patients with chemotherapy-refractory melanoma.^18,25^ Here, we report the results from a single-arm, open-label, phase IB study evaluating the safety and efficacy of axitinib in combination with toripalimab in patients with chemotherapy-naïve advanced mucosal melanoma.

## PATIENTS AND METHODS

### Patients and Study Design

This study was a phase IB, single-center, open-label, two-part (part A involved dose escalation, and part B involved cohort expansion) clinical trial (ClinicalTrials.gov identifier: NCT03086174) evaluating the safety and clinical activity of axitinib in combination with toripalimab in patients with advanced mucosal melanoma. The study was approved by the Peking University Cancer Hospital institutional review board and was conducted in accordance with the Declaration of Helsinki and Good Clinical Practice. Each patient provided written informed consent.

### Patient Eligibility

Eligible patients with pathologically confirmed metastatic melanoma must have had at least one measurable lesion per Response Evaluation Criteria in Solid Tumors (RECIST) version 1.1 at baseline, with an Eastern Cooperative Oncology Group (ECOG) performance status of 0 or 1 and adequate organ and bone marrow function. Exclusion criteria included history of autoimmune diseases; ongoing infections; or prior anti–PD-1, anti–programmed death ligand-1 (PD-L1), or anti–PD-L2 immunotherapy.

### Treatment and End Points

The planned cohorts in part A were axitinib 5 mg twice a day plus toripalimab 1 or 3 mg/kg every 2 weeks. A minimum of three patients were initially enrolled at the first dose level. If a dose-limiting toxicity occurred, then the cohort would be expanded to a total of six patients (Appendix, online only). Responses were evaluated by investigators using both RECIST version 1.1 and Immune-Related RECIST (irRECIST). Patients with progressive disease or an intolerant toxicity were taken off the study. Patients who initially developed progressive disease per RECIST version 1.1 were allowed to continue therapy if the investigator considered patients to be benefiting from the treatment per irRECIST. Any dose-escalation cohort that did not exceed the maximum-tolerated dose could be expanded in part B for additional evaluation of safety and clinical activity. The primary end point of this study was dose-limiting toxicity within the first 4 weeks of treatment with toripalimab plus axitinib in part A. The secondary end points included adverse events (graded by the National Cancer Institute Common Terminology Criteria for Adverse Events version 4.03), pharmacokinetic (PK) profile of toripalimab in the combination study, ORR, disease control rate, duration of response, PFS, OS, and status of PD-L1 and other biomarkers.

### PD-L1 Expression Analysis in Tumor Biopsies

A tumor biopsy sample was obtained for each patient before treatment initiation. PD-L1 expression was detected by immunohistochemistry staining with SP263 antibody using a Ventana (Tucson, AZ) autostainer.^26^ PD-L1 expression was evaluated on tumor cells and on tumor-infiltrating immune cells by certified pathologists. PD-L1–positive status was defined as the presence of membrane staining of any intensity in 1% or more of tumor cells or the presence of PD-L1 staining of any intensity in tumor-infiltrating immune cells covering 1% or more of tumor area occupied by tumor cells, associated intratumoral cells, and contiguous peritumoral stroma.

### TMB Analysis

Whole-exome sequencing was performed using the SureSelect Human All Exon V6 kit (Agilent, Santa Clara, CA) on tumor biopsies and matched peripheral-blood mononuclear cell samples. Genomic alterations, including microsatellite stability status, single base substitutions (single nucleotide variants), short and long insertions/deletions (indels), copy number variants, and gene rearrangement and fusions, were assessed. The TMB was determined by analyzing somatic mutations, including coding base substitution and indels per megabase.

### Messenger RNA Expression Profile Analysis

RNA was extracted from unstained formalin-fixed paraffin-embedded sections, and complementary DNA synthesis was performed followed by sequencing on the NovaSEquation 5000/6000 platform (Illumina, San Diego, CA). The relative abundance of each annotated transcript was expressed as transcripts per million and log_2_ transformed before analysis. A 12-gene panel covering inflammation and angiogenesis markers was used to generate an efficacy prediction model by logistic regression. Briefly, the abundance of RNA transcripts (transcripts per million) of selected genes was loaded into a logistic regression model to best fit coefficients to achieve the best receiver operating characteristic performance. To obtain a single score for the signature for each sample, the mean expression of the genes composing the signature was calculated.

### Statistical Analysis

Safety and efficacy analyses included all patients who received one or more dose of study medication in either arm. The ORR and its 95% exact CI were determined by the Clopper and Pearson method. PFS and OS were plotted using the Kaplan-Meier method, with medians and corresponding two-sided 95% CIs reported. Statistics analyses were performed using SAS version 9.4 (SAS Institute, Cary, NC) or GraphPad Prism software (GraphPad Software, San Diego, CA).

## RESULTS

### Patient Population

**FIG A1.**
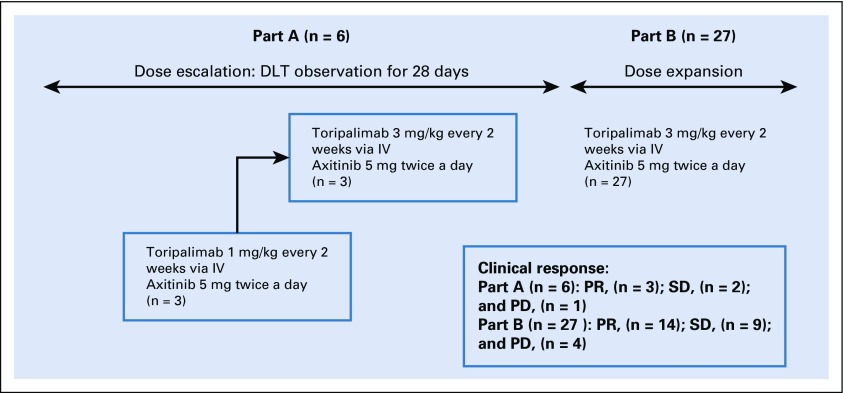
Design of phase IB combination study of toripalimab plus axitinib in treating patients with mucosal melanoma and clinical efficacy summary. DLT, dose-limiting toxicity; IV, intravenous; PD, progressive disease; PR, partial response; SD, stable disease.

**FIG A2.**
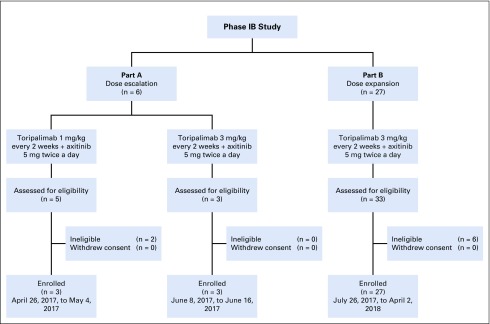
CONSORT diagram for phase IB combination study of toripalimab with axitinib in treating patients with mucosal melanoma.

From April 25, 2017, to April 2, 2018, a total of 33 patients with advanced mucosal melanoma were enrolled in the study (Appendix Figs A1 and A2, online only). Baseline characteristics are listed in Table 1. The majority of patients (31 of 33 patients) were naïve to systemic chemotherapy.

**TABLE 1.**
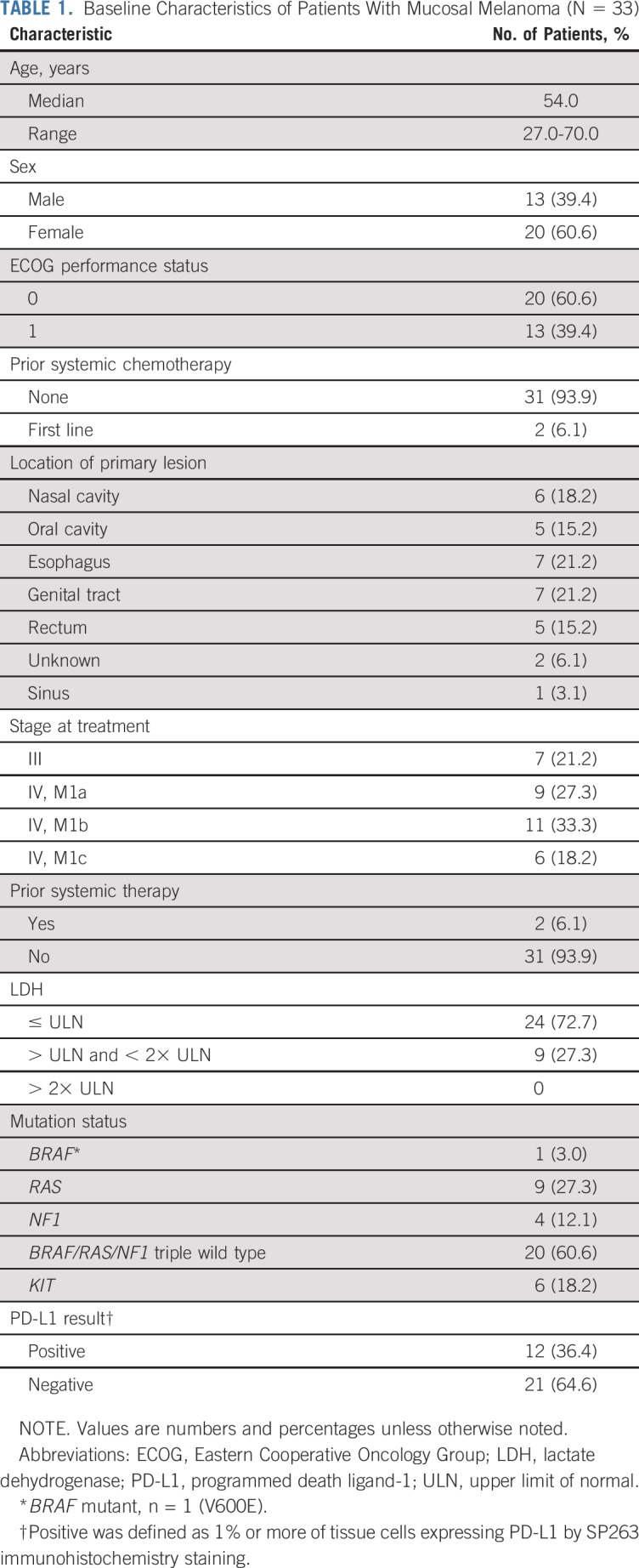
Baseline Characteristics of Patients With Mucosal Melanoma (N = 33)

### Treatment-Related Toxicity

The combination of toripalimab and axitinib was well tolerated, and no dose-limiting toxicities were observed in the initial six patients in the dose-finding phase. Subsequently, 27 patients were treated in the cohort-expansion phase (Appendix Fig A1). By December 19, 2018, 8.6 months after the last patient was enrolled, patients had received three to 42 doses of toripalimab. Thirty-two (97%) of 33 patients experienced treatment-related adverse events (TRAEs), but most were grade 1 or 2, as listed in Table 2. There were no treatment-related deaths. Grade 3 or greater TRAEs occurred in 13 patients (39.4%), including one grade 4 TRAE (lipase elevation) and 12 grade 3 TRAEs. Grade 3 TRAEs included proteinuria (n = 3), hypertension (n = 3), neutropenia (n = 3), ALT elevation (n = 2), weight loss (n = 2), diarrhea (n = 1), creatine kinase elevation (n = 1), AST elevation (n = 1), lipase elevation (n = 1), leukopenia (n = 1), anemia (n = 1), γ-glutamyl transferase elevation (n = 1), blood creatinine elevation (n = 1), hyponatremia (n = 1), and esophageal fistula (n = 1). Immune-related adverse events are listed in Table 3.

**TABLE 2.**
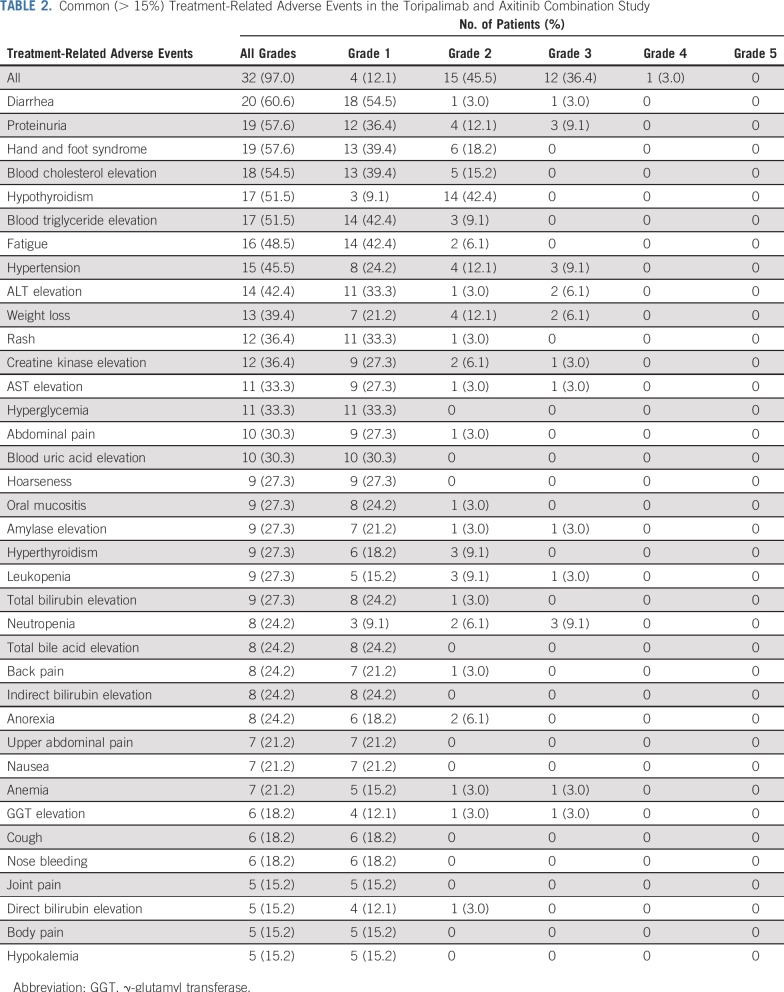
Common (> 15%) Treatment-Related Adverse Events in the Toripalimab and Axitinib Combination Study

**TABLE 3.**
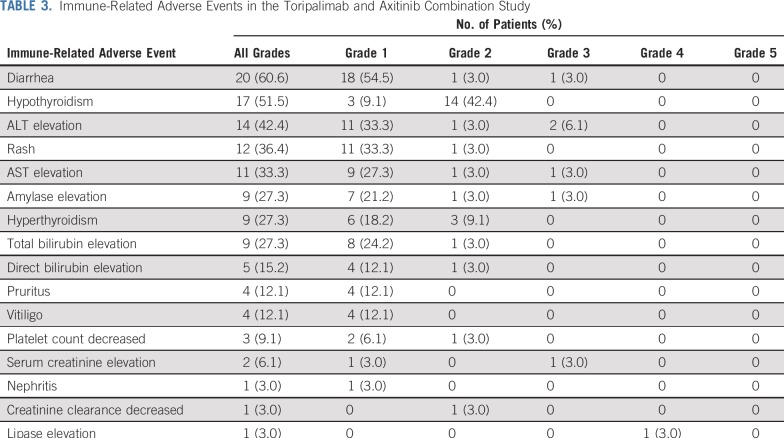
Immune-Related Adverse Events in the Toripalimab and Axitinib Combination Study

According to the protocol, the dosage of toripalimab was not permitted to be adjusted but could be delayed as a result of adverse events. Toripalimab was delayed in 10 patients. The dosage of axitinib in our study was fixed at 5 mg twice a day without dose escalation, but it could be reduced or delayed. Axitinib was delayed in 17 patients. In five patients, the dosage of axitinib was reduced to 5 mg once a day. Only one patient discontinued treatment with toripalimab and axitinib as a result of grade 3 dysphagia caused by compression from bulk in the neck. Two patients used corticosteroids to treat immune-related adverse events (one uveitis and one acute kidney injury).

**FIG A3.**
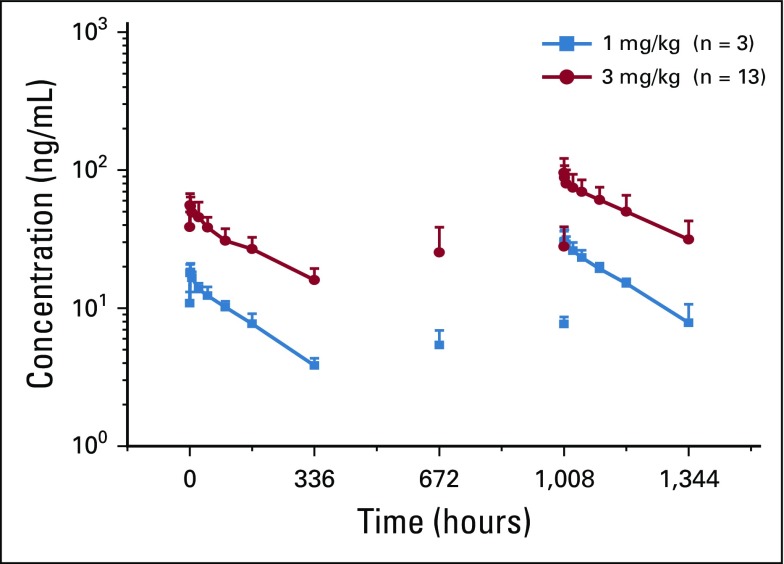
Serum concentration of toripalimab over 56 days in the combination study.

Axitinib had no apparent effect on PK parameters of toripalimab when compared with toripalimab monotherapy.^25^ The PK profile showed a dose-dependent linear exposure of toripalimab, with an elimination half-life of 8 to 16 days in the combination study (Appendix Fig A3, online only).

### Antitumor Activity

**FIG 1.**
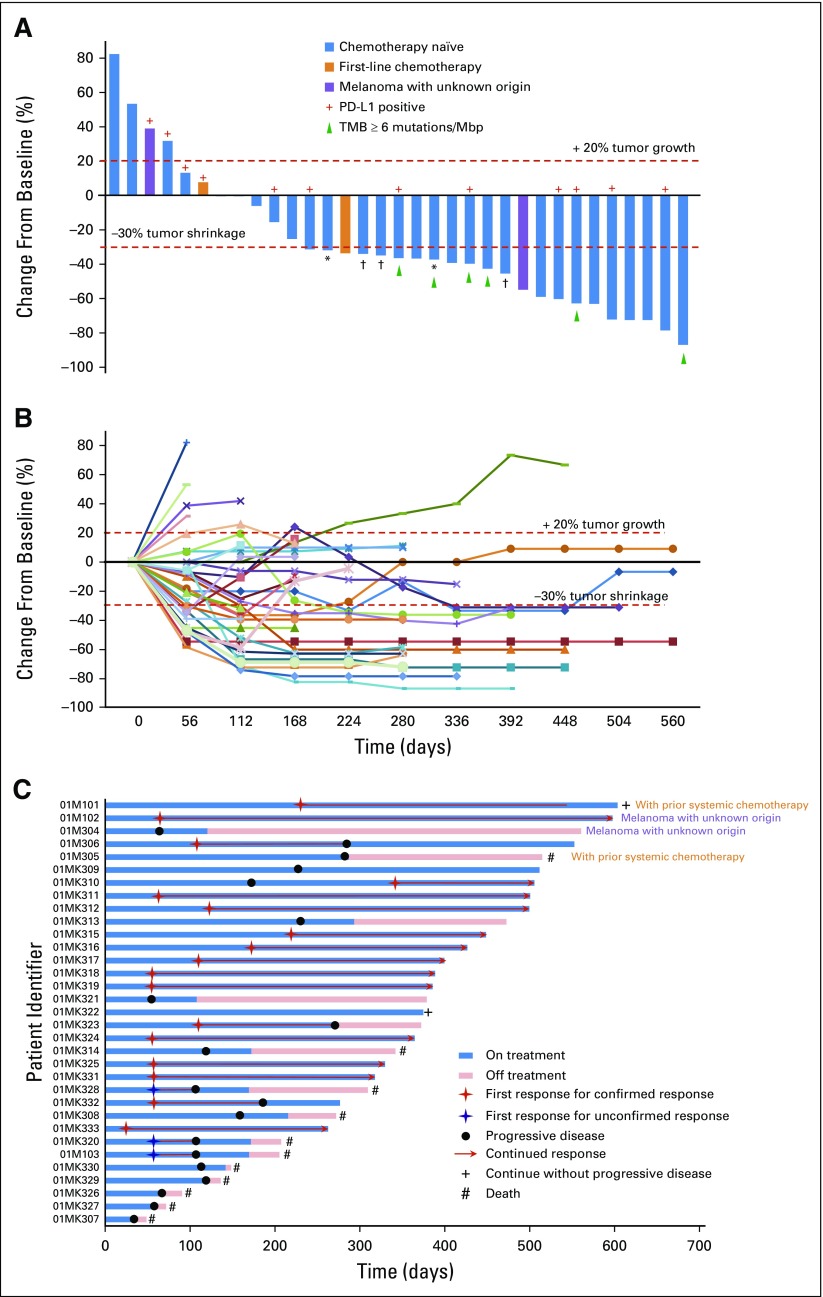
(A) Maximal change of tumor size from baseline assessed by investigator per Response Evaluation Criteria in Solid Tumors (RECIST) version 1.1 (N = 33). The length of the bar represents maximal decrease or minimal increase in target lesion(s). (B) Change in individual tumor burden over time from baseline assessed by investigator per RECIST version 1.1 (N = 33). (C) Exposure and duration of response per RECIST version 1.1 (N = 33). (*) Patient with target lesion(s) reduction of more than 30% but with new lesion(s) or progression of nontarget lesion(s). (†) Unconfirmed partial response classified as stable disease. Mbp, million base pairs; PD-L1, programmed death ligand-1; TMB, tumor mutational burden.

**FIG 2.**
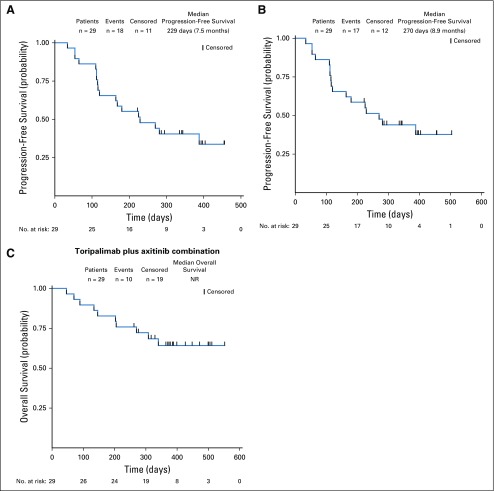
Progression-free survival by (A) Response Evaluation Criteria in Solid Tumors (RECIST) version 1.1 and (B) Immune-Related RECIST (irRECIST) and (C) overall survival of 29 patients with chemotherapy-naïve mucosal melanoma. Probability of survival is shown at indicated time points. Censored patients are marked with a vertical line in the graph. Numbers of patients at risk at indicated time points are shown below the *x*-axis. NR, not reached.

By December 19, 2018, 11 patients (33.3%) had died, four patients (12.1%) had discontinued treatment as a result of disease progression, and 18 patients (54.5%) remained on study. The median treatment duration was 9.4 months (range, 1.1 to 19.8 months). A decrease in target lesions of any size from baseline was observed in 25 patients (75.8%; Fig 1). Among 29 chemotherapy-naïve patients with mucosal melanoma assessed by investigator according to RECIST version 1.1, 14 patients (48.3%; 95% CI, 29.4% to 67.5%) achieved confirmed objective responses (complete or partial response). The disease control rate was 86.2% (95% CI, 68.3% to 96.1%). The ORR per irRECIST was 51.7% (95% CI, 32.5% to 70.6%). Three patients with stable disease had partial responses initially, but the responses were unable to be confirmed as a result of progressive disease. The median time to response was 2.1 months. The median duration of response was not reached, because 11 of 14 patients had ongoing responses. The median PFS time was 7.5 months (95% CI, 3.7 months to not evaluable) per RECIST version 1.1 and 8.9 months (95% CI 3.7 to not reached) per irRECIST. The median OS was not reached by the cutoff date (Fig 2). Notably, the clinical response was not correlated with either ECOG performance status or baseline lactate dehydrogenase levels (Data Supplement).

### PD-L1 Expression in Tumor

**FIG 3.**
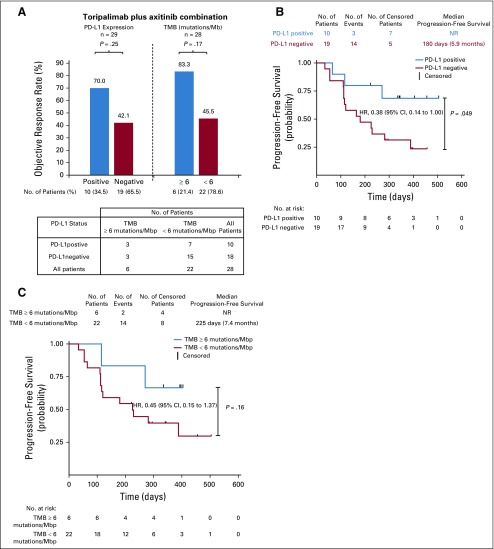
Clinical response in relation to tumor programmed death ligand-1 (PD-L1) expression and tumor mutational burden (TMB) in patients with chemotherapy-naïve mucosal melanoma. (A) PD-L1–positive status was defined as the presence of membrane staining of any intensity in 1% or more of tumor cells or immune cells by SP263 immunohistochemistry (IHC) staining. TMB was calculated by summing up somatic mutations within the coding regions by whole-exon sequencing. A TMB of 6 mutations per million base pairs (Mbp) was the cutoff value. (B) Progression-free survival per Immune-Related Response Evaluation Criteria in Solid Tumors (irRECIST) of PD-L1–positive and PD-L1–negative patients. (C) Progression-free survival per irRECIST of patients with TMB of 6 mutations/Mbp or greater and patients with TMB of less than 6 mutations/Mbp. PD-L1–positive status was defined as the presence of membrane staining of any intensity in 1% or more of tumor cells or immune cells by SP263 IHC staining. Probability of survival is shown at indicated time points. Censored patients are marked with a vertical line in the graph. Numbers of patients at risk at indicated time points are shown below the *x*-axis. HR, hazard ratio; NR, not reached.

Tumor biopsy samples were obtained from all 29 patients with chemotherapy-naïve mucosal melanoma. Ten PD-L1–positive samples (34.5%) and 19 PD-L1–negative samples (65.5%) were identified by SP263 immunohistochemistry staining.^26^ PD-L1–positive patients had a better ORR than PD-L1–negative patients to toripalimab plus axitinib combination therapy (ORR per irRECIST, 70.0% *v* 42.1%, respectively), but the difference was not statistically significant (*P* = .25; Fig 3). PD-L1–positive patients had a statistically significant PFS advantage compared with PD-L1–negative patients per irRECIST (hazard ratio, 0.38; 95% CI, 0.14 to 1.00; *P* = .049; Fig 3B).

### TMB

**FIG A4.**
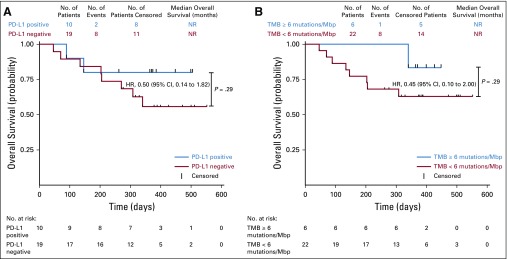
Kaplan-Meier plots of median overall survival of (A) Patients who are PD-L1 positive versus PD-L1 negative. (B) TMB ≥ 6 mutations/Mbp versus TMB < 6 mutations/Mbp patients. HR, hazard ratio; Mbp, million base pairs; NR, not reached; PD-L1, programmed death ligand-1; TMB, tumor mutational burden.

Whole-exome sequencing was performed on both tumor biopsies and paired peripheral-blood mononuclear cells from enrolled patients. Valid results were obtained from 28 patients with chemotherapy-naïve mucosal melanoma (Data Supplement). TMB was determined by analyzing somatic mutations within the coding region of the human genome. TMB was generally low in patients with mucosal melanoma in this study, with no patients with TMB of greater than 20 mutations per million base pairs (Mbp), three patients harboring more than 12 mutations/Mbp, and six patients with more than 6 mutations/Mbp. A cutoff of the top 20% of TMB in this study (6 mutations/Mbp) was selected, as suggested by Samstein et al^27^ after a correlation study of TMB value with survival in multiple cancer types. Patients with TMB of greater than 6 mutations/Mbp (n = 6) had a better ORR than patients with TMB of less than 6 mutations/Mbp (n = 22; ORR per irRECIST, 83.3% *v* 45.5%, respectively), but the difference was not statistically significant (*P* = .17). All six patients with TMB of greater than 6 mutations/Mbp had a maximum reduction of target lesion(s) of greater than 30%. However, one of the six patients had emergence of new lesions and thus did not achieve a partial response (Fig 1A). The group of patients with TMB of 6 mutations/Mbp or greater also demonstrated better PFS and OS, but the difference was not statistically significant (Fig 3C and Appendix Fig A4, online only). Notably, the subgroups with TMB of 6 mutations/Mbp or greater (n = 6) and PD-L1–positive status (n = 10) were independent in this study, because only three of 10 PD-L1–positive patients also had a TMB of 6 mutations/Mbp or greater (Fig 3A).

### Messenger RNA Expression Profile Analysis in Tumor Biopsies

**FIG 4.**
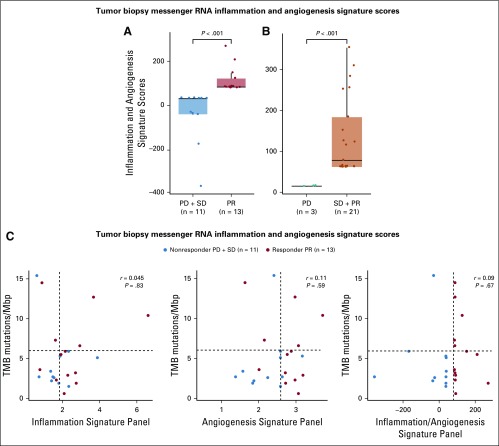
Inflammatory signature and correlation with clinical response. RNA sequencing and expression profiling of tumor biopsies were acquired from 24 patients with chemotherapy-naïve mucosal melanoma. The expression signatures of six inflammation-related genes (*IL-6*, *CXCL1*, *CXCL2*, *CXCL3*, *CXCL8*, and *PTGS2*), six angiogenesis-related genes (*VEGFA*, *KDR*, *ESM1*, *PECAM1*, *ANGPTL4*, and *CD34*), or 12 selected immune- or angiogenesis-related genes (*CD274/PD-L1*, *CXCR6*, *CD27*, *CXCL9*, *IDO1*, *TIGIT*, *PDCD1LG2/PD-L2*, *LAG3*, *ANGPTL5*, *ANGPTL6*, *CD34*, and *KDR*) were evaluated. To obtain a single score for the signature for each sample, the mean expression of the genes composing the signature was calculated. (A) The scores between patients with objective response (partial response [PR]; n = 13) per Immune-Related Response Evaluation Criteria in Solid Tumors (irRECIST) and patients with stable disease (SD) or progressive disease (PD; n = 11) were compared (*P* < .001). (B) The scores between patients achieving clinical benefit (PR or SD; n = 21) and patients with PD (n = 3) were compared (*P* < .001). (C) Associations between tumor mutational burden (TMB) and inflammation and angiogenesis gene expression profiles were also evaluated by Spearman correlation. Median TMB and median gene expression profiling scores are shown as dashed lines in the figure. *r* and *P* values of the correlations are provided. Mbp, million base pairs.

**FIG A5.**
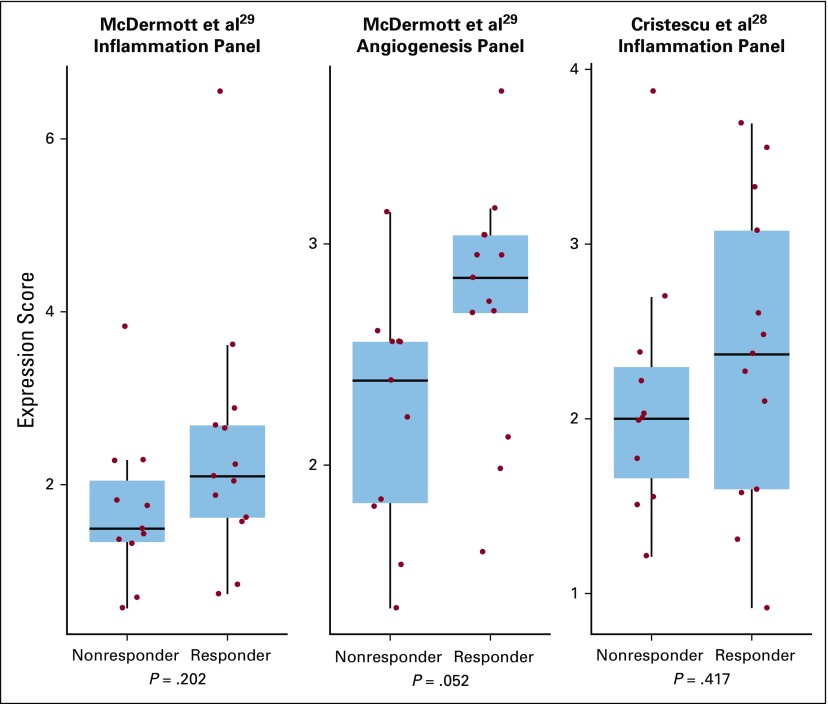
The correlation of clinical response with inflammation or angiogenesis signature panel scores. McDermott et al.^29^ inflammation panel: IL-6, CXCL1, CXCL2, CXCL3, CXCL8, and PTGS2; McDermott et al.^29^ angiogenesis panel: VEGFA, KDR, ESM1, PECAM1, ANGPTL4, and CD34; Cristescu et al.^28^ inflammation panel: IDO1, CXCL10, CXCL9, HLADRA, STAT1, and IFN-g. Responders are those with complete or partial response; nonresponders are those with stable or progressive disease.

RNA sequencing and expression profiling were performed on messenger RNA extracted from tumor biopsies. Valid results were obtained from 24 patients with chemotherapy-naïve mucosal melanoma. The 12-gene expression signatures of eight selected immune-related genes (*CD274/PD-L1*, *CXCR6*, *CD27*, *CXCL9*, *IDO1*, *TIGIT*, *PDCD1LG2/PD-L2*, and *LAG3*) and four angiogenesis-related genes (*ANGPTL5*, *ANGPTL6*, *CD34*, and *KDR*) were evaluated (Data Supplement). To obtain a single score for the signature for each sample, the mean expression of the genes composing the signature was calculated. There were statistically significant differences between the inflammatory signature scores from patients with clinical benefit (partial response plus stable disease) and patients with progressive disease per irRECIST (*P* < .001), as well as between patients with objective response (partial response) and patients without objective response (stable disease or progressive disease; *P* < .001; Figs 4A and 4B). Associations between TMB and inflammation or angiogenesis gene expression profiles (GEPs) were also evaluated using the Spearman correlation.^28^ TMB showed no association with GEP scores of angiogenesis,^29^ inflammation,^29^ or 12-gene expression signatures of inflammation or angiogenesis (Fig 4C and Appendix Fig A5, online only). Thus, GEP and TMB were independent predictors of response to the combination therapy.

### Other Biomarkers and Subgroups Analysis

**FIG A6.**
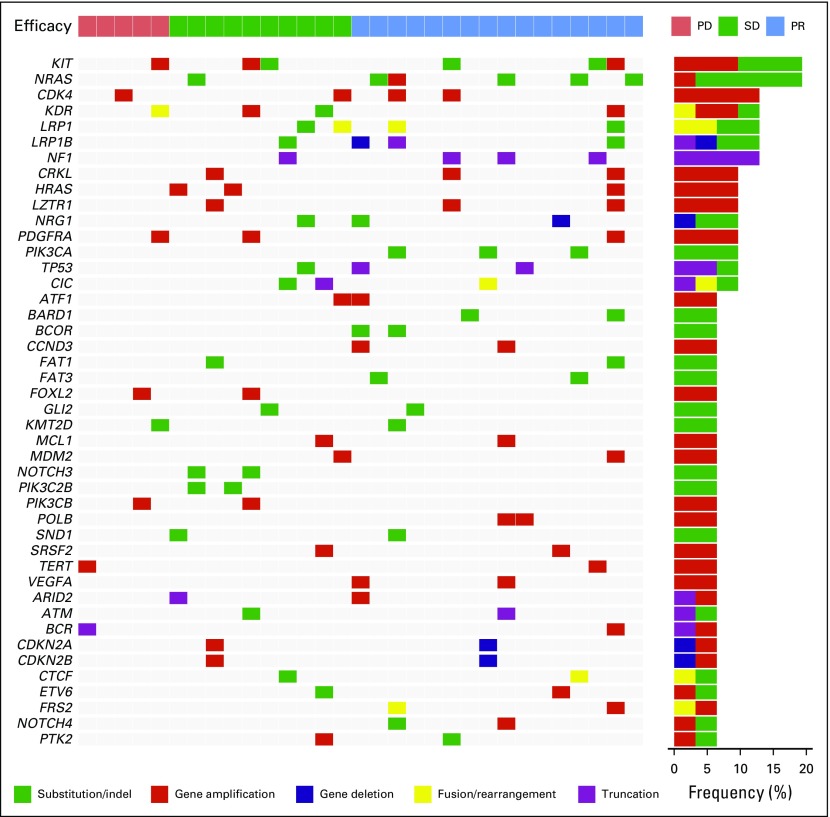
Genetic alternations and frequencies identified by whole exome sequencing (WES) from 31 available patients. Patients were grouped by clinical responses. PD, progressive disease; PR, partial response; SD, stable disease.

Whole-exome sequencing identified 5,515 genetic alternations from 31 available patients, including 127 missense, 2,642 nonsense, 299 splice site, and 68 frameshift mutations and 2,379 amplifications (Appendix Fig A6, online only). After excluding genes frequently mutated in public exomes, the top 10 most frequently altered genes were *KIT* (19%), *NRAS* (19%), *CDK4* (13%), *KDR* (13%), *LRP1* (13%), *LRP1B* (13%), *NF1* (13%), *CRKL* (10%), *LZTR1* (10%), and *NRG1* (10%). However, no correlation between mutation or pathway alteration and clinical response was found (Appendix Fig A6).

Additional biomarkers or subgroups analyzed for correlation with clinical efficacy per irRECIST included age, sex, and tumor metastatic stage (Data Supplement). Among the subgroups, those with cancer at early stages (stage III or stage IV, M1a) and esophagus as the primary site had numerically better clinical response than the rest of the patients. However, none of the differences were statistically significant.

## DISCUSSION

Mucosal melanomas have a more aggressive natural disease history than other pathologic subtypes (5-year survival rate, 26.8% *v* 53.9%, respectively).^6^ However, because of their rarity, they are less well studied; therefore, no well-established therapeutic guideline for the treatment of mucosal melanomas exists. Commonly, patients are treated with the same regimens used for cutaneous melanoma despite data suggesting they may be less effective.^6,30^

The treatment landscape for metastatic cutaneous melanoma has been greatly reshaped within the past decade as a result of two major breakthroughs, namely targeted therapy and immunotherapy.^31^ However, whether these therapeutic modalities will bring substantial benefits to mucosal melanoma is still unclear, because current available data specifically for this subtype are mostly on the basis of anecdotal case reports and retrospective analyses with small sample sizes. In terms of targeted therapy, because *BRAF* mutations occur at a much lower rate in mucosal melanomas than in cutaneous melanomas, the applicability of BRAF inhibitor–based therapy is limited.^8^ *c-Kit* mutations occur in approximately 15% of mucosal melanomas but are associated with a response rate of 35% to c-Kit inhibitors.^32,33^ As for immunotherapy, prior reports have shown that for patients with mucosal melanoma treated with ipilimumab, the ORR ranges from 7% to 12% and median PFS ranges from 2.3 to 4.3 months.^34-36^ A recent pooled analysis also showed that among patients with melanoma who received immunotherapy with nivolumab alone or in combination with ipilimumab, the ORRs were 23.3% and 37.1%, respectively, and the median PFS times were 3.0 and 5.9 months, respectively, in the mucosal subgroup.^37^ These results compare unfavorably to nivolumab monotherapy or the nivolumab plus ipilimumab combination in cutaneous melanoma (ORR, 40.9% and 60.4%, respectively; median PFS, 6.2 and 11.7 months, respectively).^37^

Here, we report the preliminary safety and efficacy results of the combination of PD-1 blockade with a VEGFR small-molecule inhibitor in patients with chemotherapy-naïve mucosal melanoma, which demonstrate a manageable safety profile and durable antitumor activity. High response rates (48.3% ORR per RECIST version 1.1) and prolonged median PFS were observed. These are encouraging findings, especially in a subtype known for its resistance to traditional chemotherapy, antiangiogenic therapy, and immunotherapy alone.

We also evaluated the predictive values of tumor PD-L1 expression, TMB, and inflammation and angiogenesis expression signatures. Although not statistically significant, PD-L1 expression and higher TMB were associated with higher ORR, consistent with previous reports in cutaneous melanomas.^38,39^ Notably, PD-L1–positive patients had a statistically significant PFS advantage compared with PD-L1–negative patients (hazard ratio, 0.38; 95% CI, 0.14 to 1.00; *P* = .049). The top 20% TMB value (6 mutations/Mbp) was used as the TMB cutoff in this study. There was no statistically significant difference in ORR for patients with 12 or more mutations/Mbp compared with 6 or more mutations/Mbp.

Messenger RNA expression signatures are known to be associated with clinical benefits. We first compared three published signatures with clinical outcomes, including an inflammation signature (*IL-6*, *CXCL1*, *CXCL2*, *CXCL3*, *CXCL8*, and *PTGS2*),^29^ angiogenesis signature (*VEGFA*, *KDR*, *ESM1*, *PECAM1*, *ANGPTL4*, and *CD34*),^29^ and interferon gamma signature (*IDO1*, *CXCL10*, *CXCL9*, *HLA-DRA*, *STAT1*, and *IFN*-γ).^40^ None of the expression signature scores were significantly different between responders (complete or partial response) and nonresponders (stable disease or progressive disease) in this study (Appendix Fig A5). Notably, angiogenesis signature alone came close to being able to differentiate responders from nonresponders statistically (*P* = .052). When compared with the inflammatory signatures, the angiogenesis signature had a stronger discriminatory power and might be broadly used as a relevant biomarker for VEGF plus immuno-oncology combination treatments. Consistently, axitinib seems to be crucial for the observed synergistic benefit in the combination study because toripalimab alone showed a 0% ORR in patients with mucosal melanoma refractory to prior chemotherapy.^18^ In this study, three short-lived unconfirmed responses were consistent with a tyrosine kinase inhibitor–induced clinical response. Because mucosal melanoma with low mutational burden might compromise the predictability of these signatures, a panel developed to include genes involved in both immune regulation and inflammation and angiogenesis might be more suitable to predict clinical response of this combination therapy. Thus, a 12-gene expression signature of eight genes related to immune regulation or inflammation (*CD274/PD-L1*, *CXCR6*, *CD27*, *CXCL9*, *IDO1*, *TIGIT*, *PDCD1LG2/PD-L2*, and *LAG3*) and four angiogenesis-related genes (*ANGPTL5*, *ANGPTL6*, *CD34*, and *KDR*) was selected to construct a logistic regression model to differentiate patients with differential clinical efficacy. However, additional validation of the expression signature in a larger cohort is needed.

Recent clinical studies combining tyrosine kinase inhibitors of the VEGFR pathways with PD-1 checkpoint inhibitors have also shown promising clinical benefit in patients with metastatic renal cell carcinoma (RCC).^41,42^ However, the disease mechanism of RCC is drastically different from mucosal melanoma, and axitinib is an approved monotherapy in RCC. Nevertheless, the combination of PD-1 and VEGFR blockade induced durable antitumor responses in otherwise poorly immunogenic tumors with low mutational burden. It remains to be investigated whether the combination treatment has a similar mechanism in RCC and mucosal melanoma. Surprisingly, the rate of grade 3 or greater toxicity observed in this combination study (39.4%) was lower than that seen with the combinations of axitinib plus pembrolizumab (71.2%) and axitinib plus avelumab (62.9%) in RCC.^41,42^ Multiple factors might contribute to this difference, including differences in histology between mucosal melanoma and RCC; ethnicity, which may influence tolerance of VEGF therapy; and the higher dose of axitinib used in a minority of patients in the other studies. One might speculate that if a higher dose of axitinib was used in this study, then a higher incidence of toxicity and better efficacy might have been observed. It remains to be investigated whether a higher dose of axitinib in the combination would result in better OS given the likely increase in grade 3 and greater toxicities. Furthermore, only two patients (6.1%) received corticosteroids to manage immune-related adverse events, which occurred less frequently than in previous nivolumab and pembrolizumab studies.^43^

In summary, this phase IB study provides evidence for the safety and efficacy of the combination of the PD-1 antibody toripalimab with axitinib in patients with advanced mucosal melanoma. Patients may benefit from the combination therapy regardless of lactate dehydrogenase level and ECOG status. Patients with PD-L1–positive tumor biopsies showed significantly better PFS than patients with PD-L1–negative biopsies. Patients with high TMB (top 20%) might also preferentially benefit from the combination treatment. Most of the clinical benefit observed in this combination study was in the stage III or IV M1a population, although the response rates in the M1b and M1c populations were also robust (> 30%). The results of our analyses, pending mature OS data, suggest that toripalimab combined with axitinib is a promising treatment option for advanced mucosal melanoma. All of the patients enrolled in this study were Asian, and the combination therapy of toripalimab and axitinib must be validated in a randomized phase III trial that includes a non-Asian population before it becomes a standard of care for patients with advanced mucosal melanoma.

## Appendix

### Definition of Dose-Limiting Toxicity

Dose-limiting toxicity was classified as any of the following: grade 4 neutropenia or thrombocytopenia, grade 3 or worse neutropenic infection or thrombocytopenia with bleeding or febrile neutropenia; nonhematologic grade 3 or worse toxicity; and inability to complete at least 75% of axitinib dosing or two infusions of toripalimab as a result of treatment-related toxicity occurring during the 4-week observation period for dose-limiting toxicities and attributable to one or both study drugs.

### Messenger RNA Expression Profile Analysis

RNA was extracted from unstained formalin-fixed paraffin-embedded sections using the miRNeasy FFPE Kit (catalog No. 217504; Qiagen, Venlo, the Netherlands) and was further processed with a NEBNext rRNA Depletion Kit (catalog No. E6310L; New England Biolabs, Ipswich, MA) to remove ribosomal RNA. First-strand and second-strand cDNA synthesis were performed using M-MLV RT RNase(H-) (catalog No. M3683; Promega, Madison, WI) and NEB Second Strand Messenger RNA Synthesis Kit (catalog No. E6111L; New England Biolabs), respectively. The complementary DNA product was sonicated to produce an approximately 200–base pair fragment size (E220; Covaris, Woburn, MA). Adaptor-ligated libraries were created from the complementary DNA using a KAPA Hyper Prep Kit (catalog No. 07962363001; Roche, Basel, Switzerland) for sequencing on the NovaSEquation 5000/6000 platform (Illumina, San Diego, CA).. The relative abundance of each annotated transcript was expressed as transcripts per million and log_2_ transformed before analysis. A 12-gene panel covering inflammation (*CD274*, *CXCR6*, *CD27*, *CXCL9*, *IDO1*, *TIGIT*, *PDCD1LG2*, and *LAG3*) and angiogenesis (*ANGPTL5*, *ANGPTL6*, *CD34*, and *KDR*) markers was used to generate an efficacy prediction model by logistic regression. Briefly, the abundance of RNA transcript (transcripts per million) of selected genes was loaded into a logistic regression model to best fit coefficients to achieve the best receiver operating characteristic performance. To obtain a single score for the signature for each sample, the mean expression of the genes composing the signature was calculated.

### Sample Size Determination

The sample size for the dose-finding phase could not be determined in advance of the study because of the unknown safety profile of combined toripalimab and axitinib therapy. In part A (dose escalation) of this study, three of six patients achieved a partial response, and the objective response rate (ORR) was 50%. We estimated the sample size on the basis of the following assumptions: 50% ORR for axitinib plus toripalimab combination. On the basis of a phase II axitinib monotherapy trial in metastatic melanoma (n = 32), where an 18.8% ORR was observed (Fruehauf J, et al: Clin Cancer Res 17:7462-7469, 2011), we assumed the efficacy for axitinib monotherapy is approximately 20%. At a two-sided significance level of *P* = .05, a total of 30 patients could provide 90% power to show the efficacy of toripalimab in combination with axitinib when the targeted ORR of the combination therapy is 50% compared with 20% for axitinib monotherapy. Assuming a dropout rate of 10%, an initial 33-patient enrollment was planned. Thus, we decided to enroll 27 additional patients in the dose-expansion group.
